# Soziale Lage, Gesundheit und Gesundheitsverhalten von Kindern und Jugendlichen in Ein-Eltern-Haushalten zum Ende der COVID-19-Pandemie. Ergebnisse der KIDA-Studie 2022–2023

**DOI:** 10.1007/s00103-024-03910-9

**Published:** 2024-07-12

**Authors:** Petra Rattay, Yasmin Öztürk, Raimund Geene, Miriam Blume, Jennifer Allen, Christina Poethko-Müller, Elvira Mauz, Kristin Manz, Catherine Wieland, Claudia Hövener

**Affiliations:** 1 Abteilung Epidemiologie und Gesundheitsmonitoring, Robert Koch-Institut, General-Pape-Str. 62–66, 12101 Berlin, Deutschland; 2 Institut für Medizinische Soziologie, Medizinische Fakultät und Universitätsklinikum Düsseldorf, Heinrich-Heine-Universität Düsseldorf, Düsseldorf, Deutschland; 3 Abteilung Familie und Familienpolitik, Deutsches Jugendinstitut, München, Deutschland; 4 Berlin School of Public Health, Alice Salomon Hochschule, Berlin, Deutschland; 5 Berliner Institut für Gesundheits- und Sozialwissenschaften, Europa-Institut für Sozial- und Gesundheitsforschung, Berlin School of Public Health, Berlin, Deutschland

**Keywords:** Kinder- und Jugendgesundheit, Familie, Alleinerziehende, Familienform, Gesundheitliche Ungleichheit, Children’s and adolescents’ health, Family, Single-parent family, Family type, Health inequality

## Abstract

**Einleitung:**

In der COVID-19-Pandemie waren Alleinerziehende und ihre Kinder durch die Eindämmungsmaßnahmen und aufgrund oftmals geringer Ressourcen in besonderem Maße Belastungen ausgesetzt. Es wird analysiert, inwieweit sich zum Ende der Pandemie Unterschiede in der sozialen und gesundheitlichen Lage von Kindern und Jugendlichen in Ein-Eltern- und Zwei‑Eltern-Haushalten zeigen.

**Methoden:**

Die Analyse basiert auf Daten der KIDA-Studie, in der 2022/2023 Eltern von 3‑ bis 15-Jährigen und 16- bis 17-Jährige befragt wurden (telefonisch: *n* = 6992; online: *n* = 2896). Für die Indikatoren psychosoziale Belastungen, soziale Unterstützung, Gesundheit und Gesundheitsverhalten wurden nach Familienform stratifizierte Prävalenzen berechnet. In Poisson-Regressionen wurde für Geschlecht, Alter, Bildung und Haushaltseinkommen adjustiert.

**Ergebnisse:**

Heranwachsende aus Ein-Eltern-Haushalten sind häufiger durch finanzielle Einschränkungen, familiäre Konflikte und beengte Wohnverhältnisse belastet und erfahren weniger schulische Unterstützung als Gleichaltrige aus Zwei‑Eltern-Haushalten. Sie haben häufiger gesundheitliche Beeinträchtigungen sowie einen erhöhten Versorgungsbedarf und nehmen häufiger psychosoziale Angebote in Anspruch. Sie sind zwar seltener in Sportvereinen aktiv, nehmen jedoch gleich häufig an Sport-AGs in Schulen teil wie Gleichaltrige aus Zwei-Eltern-Haushalten. Die Unterschiede zeigen sich auch bei Kontrolle für Einkommen und Bildung.

**Diskussion:**

Kinder und Jugendliche aus Ein-Eltern-Haushalten können über Bewegungsangebote im schulischen Setting gut erreicht werden. Niedrigschwellige Angebote in Kita, Schule und Kommune sollten daher weiter ausgebaut werden. Weiterhin bedarf es Maßnahmen zur Verbesserung der sozioökonomischen Lage von Alleinerziehenden und ihren Kindern.

## Abstract (en)

**Introduction:**

During the COVID-19 pandemic, single parents and their children were particularly exposed to stress due to the containment measures and to limited resources. We analyzed differences in the social and health situation of children and adolescents in one-parent households and two-parent households at the end of the pandemic.

**Methods:**

The analysis is based on data from the KIDA study, in which parents of 3‑ to 15-year-old children as well as 16- to 17-year-old adolescents were surveyed in 2022/2023 (telephone: *n* = 6992; online: *n* = 2896). Prevalences stratified by family type were calculated for the indicators psychosocial stress, social support, health, and health behavior. Poisson regressions were adjusted for gender, age, level of education, and household income.

**Results:**

Children and adolescents from one-parent households are more likely to be burdened by financial restrictions, family conflicts, and poor living conditions and receive less school support than peers from two-parent households. They are more likely to have impairments in health as well as increased healthcare needs, and they use psychosocial services more frequently. Furthermore, they are less likely to be active in sports clubs, but they take part in sporting activities at schools as often as minors from two-parent households. The differences are also evident when controlling for income and education.

**Discussion:**

Children and adolescents from one-parent households can be reached well through exercise programs in a school setting. Low-threshold offers in daycare centers, schools, and the community should therefore be further expanded. Furthermore, interventions are needed to improve the socioeconomic situation of single parents and their children.

## Einleitung

Im Jahr 2022 lebten in Deutschland 2,26 Mio. Kinder unter 18 Jahren in Haushalten mit nur einem Elternteil; in 85,6 % war dies die alleinerziehende Mutter [1]. Der Anteil der Kinder und Jugendlichen in Ein-Eltern-Haushalten an allen Kindern und Jugendlichen betrug 16,0 % [1].

Familienformen sind in den letzten Jahrzehnten diverser geworden, was auch auf die Familien von Alleinerziehenden^1^ und ihren Kindern zutrifft [2, 3]. Die Konstellationen, in denen Mütter und Väter ihre Kinder heute nach einer Trennung betreuen, reichen von der alleinigen Zuständigkeit eines Elternteils über die geteilte Betreuung bis hin zum Wechselmodell [3–5]. Im Jahr 2020 gaben jedoch nur 12 % der getrenntlebenden Eltern eine ausgewogene Aufteilung der Verantwortung für ihre Kinder an [6]. Trotz der Pluralisierung von Familienformen und einer größeren gesellschaftlichen Akzeptanz sind Familien, in denen ein Elternteil allein mit Kindern zusammenlebt, auch heute oftmals aufgrund struktureller Benachteiligungen, die u. a. in Vereinbarkeitsproblemen von Familie und Beruf zum Ausdruck kommen, sowie begrenzter zeitlicher, sozialer und finanzieller Ressourcen vor große Herausforderungen gestellt [3, 6]. Besonders deutlich wird dies an der hohen Armutsgefährdungsquote von Haushalten mit einem alleinerziehenden Elternteil, die im Jahr 2021 bei 41,6 % lag [7].

Kinder und Jugendliche können aufgrund der Trennung der Eltern, dem Tod eines Elternteils oder von Geburt an mit nur einem Elternteil zusammen in einem Haushalt leben. Die Trennung der Eltern sowie der Tod eines Elternteils werden als kritische Lebensereignisse in Kindheit und Jugend gewertet [8], die Auswirkungen auf die soziale Lebenssituation der betroffenen Kinder und Jugendlichen haben können. Hierzu zählen z. B. der Verlust oder die Einschränkung des Kontakts zu einem Elternteil, möglicherweise der Umzug in eine neue Wohnung und ein eventuell damit einhergehender Wechsel der Schule und des Freundeskreises oder finanzielle Einschränkungen. Diese Erfahrungen sowie die oftmals geringeren zeitlichen, sozialen und finanziellen Ressourcen von Alleinerziehenden können Auswirkungen auf das Wohlbefinden und die psychische und körperliche Gesundheit der Kinder haben [9].

Für Deutschland belegen für die Zeit vor der COVID-19-Pandemie verschiedene Studien, dass insbesondere die psychische Gesundheit von Kindern und Jugendlichen in Ein-Eltern-Haushalten häufiger beeinträchtigt war als jene Gleichaltriger in Zwei‑Eltern-Haushalten [10–15]. Bei der körperlichen Gesundheit finden sich nur für einzelne Indikatoren wie Asthma höhere Prävalenzen für Kinder aus Ein-Eltern-Haushalten [16]. Mit Blick auf das Gesundheitsverhalten zeigt sich, dass Kinder aus Ein-Eltern-Familien seltener in Sportvereinen aktiv waren [17].

Während der COVID-19-Pandemie waren Familien im Allgemeinen und Familien von Alleinerziehenden im Besonderen von den Eindämmungsmaßnahmen (Einschränkung der sozialen Kontakte, Kita- und Schulschließungen, Homeschooling, Homeoffice etc.) betroffen, die erhebliche Einschränkungen und Veränderungen im Leben der Kinder und Jugendlichen mit sich brachten [6, 18, 19]. Bei Kindern und Jugendlichen lässt sich als Folge dieser Belastungen insbesondere eine deutliche Verschlechterung der psychischen Gesundheit feststellen [20–24]. Zum Ende der Pandemie in 2022 nahmen psychische Beeinträchtigungen zwar ab, blieben aber dennoch auf einem höheren Niveau als vor der Pandemie [20]. Für das Bewegungsverhalten von Kindern und Jugendlichen zeigt sich insbesondere während der Schulschließungen zu Beginn der Pandemie ein deutlicher Rückgang [20, 25, 26].

Speziell zur Gesundheit von Kindern, die mit einem alleinerziehenden Elternteil zusammenleben, gibt es für Deutschland für den Zeitraum der Pandemie aber bislang nur wenige Studien. Laut COPSY-Studie (**Co**rona und **Psy**che) hatten in 2020 und Anfang 2021 Kinder und Jugendliche aus Ein-Eltern-Haushalten eine schlechtere gesundheitsbezogene Lebensqualität sowie häufiger psychische Gesundheitsprobleme und psychosomatische Beschwerden als Gleichaltrige aus Zwei‑Eltern-Haushalten [21]. In einer Umfrage unter Versicherten der AOK (Allgemeine Ortskrankenkasse) [18] im Februar/März 2022 schätzten Alleinerziehende sowohl die psychische als auch die körperliche Gesundheit ihrer 3‑ bis 12-jährigen Kinder seltener als sehr gut oder gut ein als Eltern aus Paarhaushalten. Alleinerziehende berichteten zudem für ihre Kinder häufiger eine Verschlechterung der psychischen und körperlichen Gesundheit während der Pandemie [18]. Eine auf Daten aus Einschulungsuntersuchungen beruhende Trendanalyse [27] zeigt, dass Übergewicht im Pandemiezeitraum bei Kindern von Alleinerziehenden stärker zugenommen hat als bei Kindern aus Zwei‑Eltern-Haushalten. Vor der Pandemie (2018/2019) und im letzten Pandemiejahr (2022/2023) zeigen sich hingegen keine Unterschiede in den Prävalenzen nach Familienform [27]. Insgesamt liegen für Deutschland für das Ende und die erste Zeit nach der Pandemie sowie für ältere Kinder und Jugendliche nur wenige Ergebnisse zur Gesundheit von Heranwachsenden aus Ein-Eltern-Haushalten vor. An dieser Forschungslücke setzt die vorliegende Analyse an.

Ziel des Beitrags ist es, einen Überblick über die gesundheitliche Lage von Kindern und Jugendlichen aus Ein-Eltern-Haushalten im dritten Jahr der Pandemie und nach Beendigung der Eindämmungsmaßnahmen Anfang 2023 zu geben. Dies erfolgt in vergleichender Weise zur Gesundheit von Kindern und Jugendlichen aus Zwei‑Eltern-Haushalten. Darüber hinaus werden in der Analyse Unterschiede in den sozialen Lagen sowie den psychosozialen Belastungen und Ressourcen während der Pandemie zwischen den Familienformen berücksichtigt. Da Ein-Eltern-Haushalte in höherem Maße von Armut betroffen sind, wird ein besonderer Fokus auf die Frage gelegt, ob die Zusammenhänge zwischen Familienform und Gesundheit mit dem Familieneinkommen variieren.

Im Einzelnen geht der Beitrag folgenden Fragen nach:

1. Zeigen sich in der sozialen Lage, in der Gesundheit und im Gesundheitsverhalten Unterschiede zwischen Kindern und Jugendlichen aus Ein-Eltern- und Zwei‑Eltern-Haushalten?
2. Bestehen die Unterschiede in der Gesundheit und im Gesundheitsverhalten unabhängig vom Einkommen und Bildungsstand des Haushalts?
3. Variieren die Zusammenhänge zwischen der Familienform und der Gesundheit bzw. dem Gesundheitsverhalten von Kindern und Jugendlichen mit dem Einkommen des Haushalts?

Der vorliegende Beitrag liefert Ergebnisse für den Zehnten Familienbericht, der sich unter dem Titel „Unterstützung allein- und getrennterziehender Eltern und ihrer Kinder – Bestandsaufnahme und Handlungsempfehlungen“ den vielfältigen Aspekten der Lebenssituation, einschließlich der gesundheitlichen Lage, von Allein- und Getrennterziehenden und ihren Kindern widmet [28].

## Daten und Methoden

### Daten

Die Studie „Kindergesundheit in Deutschland aktuell“ (KIDA) untersuchte im Zeitraum von Februar 2022 bis Juni 2023 die Gesundheit und das Gesundheitsverhalten von Kindern und Jugendlichen im Alter von 3–17 Jahren. Im Rahmen der Querschnittsstudie wurden Eltern mit Kindern im Alter von 3–15 Jahren und Jugendliche im Alter von 16–17 Jahren telefonisch und online mittels standardisierter Fragebögen befragt. Die Rekrutierung der Teilnehmenden erfolgte über die Studie „Gesundheit in Deutschland aktuell“ (GEDA), die die Gesundheit von Personen ab 16 Jahren erhebt [29]. Die Stichprobe der GEDA-Studie wurde über das Dual-Frame-Verfahren gezogen, bei dem 2 Auswahlrahmen, Mobilfunk- und Festnetznummern, genutzt werden [30]. In der GEDA-Studie wurden zwischen Februar 2022 und April 2023 teilnehmende Erwachsene mit einem Kind im Alter von 3–15 Jahren im Haushalt zur Teilnahme an der telefonischen KIDA-Studie eingeladen. 80 % der in der GEDA-Studie interviewten Eltern waren bereit, die Fragen zur Gesundheit ihrer Kinder zu beantworten. Dies war für maximal 2 im Haushalt lebende Kinder möglich. In Haushalten mit mehr als 2 Kindern erfolgte eine zufällige Auswahl. 16- und 17-Jährige, die an der GEDA-Studie teilnahmen, wurden direkt in dem Telefoninterview auch zu den KIDA-Inhalten befragt. Nach Abschluss des Telefoninterviews wurden alle KIDA-Teilnehmenden gebeten, zusätzlich auch an der Online-Befragung teilzunehmen, in der vertiefende bzw. weitere Inhalte erfragt wurden. Diese erfolgte von April 2022 bis Juni 2023 [31]. Die Stichprobe der telefonischen Befragung umfasst 6992 Kinder und Jugendliche. Die Online-Befragung lieferte für 2896 dieser Kinder und Jugendlichen zusätzliche Informationen.

### Variablen

Als Outcome-Variablen gingen zur Beschreibung der sozialen Lage das Einkommen der Familie, der höchste Bildungsabschluss des Haushaltes sowie mit Blick auf die Kinder und Jugendlichen psychosoziale Belastungen und die soziale Unterstützung im privaten und schulischen/betrieblichen Umfeld ein. In Bezug auf die Gesundheit und das Gesundheitsverhalten wurden der allgemeine Gesundheitszustand, die allgemeine psychische Gesundheit, ein erhöhter Versorgungs- bzw. Unterstützungsbedarf, die Teilnahme an außerschulischen Sportvereinen oder -kursen, die Teilnahme an freiwilligen Bewegungs- oder Sportangeboten (Sport-AGs) in der Schule sowie die Inanspruchnahme mindestens eines psychosozialen Angebots in den letzten 4 Wochen ausgewählt.

Die Familienform fungierte als Prädiktorvariable.

Als Kontrollvariablen wurden das Geschlecht und das Alter der Kinder und Jugendlichen, die Wohnregion und das Untersuchungsjahr berücksichtigt. Darüber hinaus gingen in die multivariaten Analysen das Haushaltseinkommen und der höchste Bildungsabschluss im Haushalt ein.

Eine ausführliche Darstellung der Variablen findet sich in Tab. 1 und die Beschreibung der Stichprobe in Tab. 2.

**Tab. 1:** Beschreibung der Variablen

Variable	Erhebungsmodus	Ergebungsinstrument	Operationalisierung der Antwortvorgaben	Quelle
Familienform	Telefon: Eltern (3–15 J.) Jugendliche (16–17 J.)	Die Familienform wird über die Zusammensetzung des Haushalts der Befragten operationalisiert. In Abhängigkeit davon, ob in dem Haushalt ein oder 2 Elternteile mit dem Kind zusammenleben, wird zwischen „Ein-Eltern-Haushalt“ und „Zwei-Eltern-Haushalt“ differenziert. Dabei wird nicht zwischen biologischer und sozialer Elternschaft unterschieden. Zwei‑Eltern-Haushalte umfassen auch sogenannte Stieffamilien. Informationen zu den Tagen, die das Kind ggf. in einem weiteren Haushalt lebt, wurden in der KIDA-Studie nicht erfasst. Aussagen explizit zu Kindern, die mit getrennterziehenden Eltern bzw. im Wechselmodell leben, sind daher nicht möglich. Weitere erwachsene Personen (z. B. Großeltern), die im Haushalt leben, wurden nicht berücksichtigt.	– Ein-Eltern-Haushalt – Zwei‑Eltern-Haushalt	[55]
Geschlecht	Telefon: Eltern (3–15 J.) Jugendliche (16–17 J.)	Eltern: *Welches Geschlecht wurde bei Ihrem Kind in die Geburtsurkunde eingetragen?* Jugendliche: *Da sich nicht alle Menschen ihrem eingetragenen Geschlecht zugehörig fühlen: Welchem Geschlecht fühlen Sie sich zugehörig?*	– Männlich – Weiblich – Divers	–
Alter	Telefon Eltern (3–15 J.) Jugendliche (16–17 J.)	Eltern: Das Alter der Kinder wurde über Fragen zur Zusammensetzung des Haushalts erfasst und danach gruppiert. Jugendliche: *In welchem Jahr/Monat sind Sie geboren?*	– 3–6 Jahre – 7–10 Jahre – 11–13 Jahre – 14–15 Jahre – 16–17 Jahre	–
Wohnregion	Telefon Eltern (3–15 J.) Jugendliche (16–17 J.)	*In welchem Bundesland wohnen Sie?*	– Neue Bundesländer – Alte Bundesländer – Berlin	–
Haushaltseinkommen	Telefon Eltern (3–15 J.) Jugendliche (16–17 J.)	Bedarfsgewichtetes Haushaltsnettoäquivalenzeinkommen	– Niedrig (< 60 % des Medians = armutsgefährdet) – Mittel (60 % bis < 150 % des Medians) – Hoch (≥ 150 % des Medians)	[56]
Bildung	Telefon Eltern (3–15 J.) Jugendliche (16–17 J.)	Höchster Bildungsabschluss des Haushaltes CASMIN-Klassifikation	– Niedrig – Mittel – Hoch	[57]
Psychosoziale Belastungen in den letzten 4 Wochen	Online Eltern (3–15 J.) Jugendliche (16–17 J.)	*Inwieweit hat sich Ihr Kind/haben Sie sich in den letzten vier Wochen durch folgende Ereignisse/Vorkommnisse belastet gefühlt?* – Finanzielle Einschränkungen – Beengte Wohnverhältnisse – Konflikte in der Familie 7‑Punkte-Skala von nicht belastet (1) bis sehr stark belastet (7) bzw. trifft nicht zu (8)	– Belastet (2–7) – Nicht belastet (1)/trifft nicht zu (8)	–
Unterstützung im schulischen und privaten Umfeld im letzten Monat	Online Eltern (6–15 J., Kind besucht Schule) Jugendliche (16–17 J. in Schule oder Ausbildung)	*Was trifft auf Ihr Kind/Sie im letzten Monat zu? Hatte Ihr Kind/Hatten Sie (zuhause) ausreichenden Zugang zu den folgenden Ressourcen oder Unterstützung?* – Hilfe/Unterstützung von LehrerInnen oder anderen Personen aus dem schulischen Umfeld – Hilfe/Unterstützung von einem Elternteil oder einer anderen Person aus dem privaten Umfeld	– Meistens/immer – Nie/selten/manchmal	–
Allgemeine Gesundheit	Telefon Eltern (3–15 J.) Jugendliche (16–17 J.)	Eltern: *Wie ist der Gesundheitszustand Ihres Kindes im Allgemeinen?* Jugendliche: *Wie ist Ihr Gesundheitszustand im Allgemeinen?*	– Sehr gut/gut – Mittelmäßig/schlecht/sehr schlecht	–
Allgemeine psychische Gesundheit	Telefon Eltern (3–15 J.) Jugendliche (16–17 J.)	Eltern: *Wie würden Sie die psychische Gesundheit Ihres Kindes im Allgemeinen einschätzen?* Jugendliche: *Wie würden Sie Ihren psychischen Gesundheitszustand im Allgemeinen beschreiben?*	– Ausgezeichnet/sehr gut – Gut/weniger gut/schlecht	[46]
Erhöhter Versorgungs- bzw. Unterstützungsbedarf	Telefon Eltern (3–15 J.) Jugendliche (16–17 J.)	*– Braucht Ihr Kind/Brauchen Sie mehr medizinische Versorgung, psychosoziale oder pädagogische Unterstützung, als es für Kinder/Jugendliche in diesem Alter üblich ist?* (Ja/Nein) – *Geschieht dies aufgrund einer Krankheit, Verhaltensstörung oder eines gesundheitlichen Problems?* (Ja/Nein) – *Dauert dieses Problem bereits 12 Monate an oder ist eine Dauer von mehr als 12 Monaten zu erwarten?* (Ja/Nein) Frage 1 des Children with Special Health Care Needs (CSHCN) Screener Ein erhöhter Versorgungs- bzw. Unterstützungsbedarf liegt vor, wenn alle 3 Fragen mit „Ja“ beantwortet wurden.	– Ja – Nein	[58]
Teilnahme an freiwilligen Bewegungs- oder Sport-AGs in der Schule in den letzten 4 Wochen	Telefon Eltern (5–15 J., wenn Schule im Regelbetrieb und keine Quarantäne)	*Denken Sie bitte nun an die letzten 4 Wochen: Hat Ihr Kind an Bewegungs- oder Sport-AGs in der Schule teilgenommen?* Hinweis: Es sind freiwillige Nachmittagsangebote gemeint.	– Ja – Nein	–
Teilnahme an Sportverein/-kurs in den letzten 4 Wochen	Telefon Eltern (3–15 J.) Jugendliche (16–17 J.)	*Denken Sie bitte nun an die letzten 4 Wochen: Hat Ihr Kind/Haben Sie an Sportvereinsangeboten oder Sportkursen in Fitnessstudios, Ballett- oder Schwimmschulen usw. teilgenommen?*	– Ja – Nein	–
Inanspruchnahme mindestens eines psychosozialen Unterstützungsangebots in den letzten 4 Wochen	Online Eltern (3–15 J.) Jugendliche (16–17 J.)	*Welche der im Folgenden genannten Unterstützungsangebote haben Sie mit oder für Ihr Kind/haben Sie in den letzten vier Wochen in Anspruch genommen, um die Herausforderungen im Zusammenhang mit der COVID-19-Pandemie besser bewältigen zu können?* – Psychosoziale Unterstützungs- und Beratungsangebote vor Ort, wie z. B. Familien- oder Eltern-Kind-Beratungsstellen, Selbsthilfe-Gruppen, Eltern-Kind-Cafés – Online-Portale mit Informationen und Tipps zur psychosozialen Gesundheit, wie z. B. „psychisch stabil bleiben“, „Corona und du“, „angstfrei.news“ oder das „Familienportal“ – Online-Trainings zur Stärkung der psychosozialen Gesundheit, wie z. B. „get.calm and move.on“, „stark durch die Krise“ – Gesundheits-Apps zur Stärkung der psychischen Gesundheit, wie z. B. „iFightDepression“ oder die „KrisenKompass App“ – Telefonische psychosoziale Unterstützung und Beratung, wie z. B. „Nummer gegen Kummer“	– Ja (mindestens 1 Angebot in Anspruch genommen) – Nein (kein Bedarf/Angebot ist nicht bekannt/andere Gründe)	–

**Tab. 2:** Beschreibung der Stichprobe

		Telefonbefragung		Online-Befragung	
	Alter	*n* (ungewichtet)	% (gewichtet)	*n* (ungewichtet)	% (gewichtet)
*Insgesamt*	3–17	6992	100,0	2894	100,0
*Familienform*	3–17				
Zwei‑Eltern-Haushalt		6164	79,4	2548	80,0
Ein-Eltern-Haushalt		788	20,6	341	20,0
Fehlende Werte		40	–	5	–
*Geschlecht*	3–17				
Männlich		3653	51,4	1490	50,2
Weiblich		3336	48,6	1404	49,8
Divers		3	0,0	0	0,0
Fehlende Werte		0	–	0	–
*Alter*	3–17				
3–6 Jahre		1724	27,9	739	28,2
7–10 Jahre		1934	25,6	826	26,1
11–13 Jahre		1639	19,9	706	19,9
14–15 Jahre		1217	13,2	489	12,5
16–17 Jahre		478	13,4	134	13,3
Fehlende Werte		0	–	0	–
*Wohnregion*	3–17				
Alte Bundesländer		5637	80,9	2355	81,3
Neue Bundesländer		950	13,8	369	14,3
Berlin		404	5,3	170	4,4
Fehlende Werte		1	–	0	–
*Untersuchungsjahr*	3–17				
2022		5031	71,6	1923	67,9
2023		1961	28,4	971	32,1
Fehlende Werte		0	–	0	–
*Einkommen (bedarfsgewichtetes Haushaltseinkommen)*	3–17				
Niedrig		814	21,9	249	17,7
Mittel		4696	65,6	2000	70,8
Hoch		1482	12,5	645	11,4
Fehlende Werte		0	–	0	–
*Bildung (höchster Bildungsabschluss im Haushalt)*	3–17				
Niedrig		235	17,5	72	17,4
Mittel		2217	50,0	840	50,5
Hoch		4506	32,5	1976	32,1
Fehlende Werte		34	–	6	–
*Finanzielle Einschränkungen in den letzten 4 Wochen*	3–17				
Ja		–	–	613	26,9
Nein		–	–	2182	73,1
Fehlende Werte		–	–	99	–
*Konflikte in der Familie in den letzten 4 Wochen*	3–17				
Ja		–	–	1171	39,5
Nein		–	–	1623	60,5
Fehlende Werte		–	–	100	–
*Beengte Wohnverhältnisse in den letzten 4 Wochen*	3–17				
Ja		–	–	369	15,4
Nein		–	–	2422	84,6
Fehlende Werte		–	–	103	–
*Unterstützung aus schulischem/betrieblichem Umfeld*	6–17				
Meistens/immer		–	–	1609	69,8
Nie/selten/manchmal		–	–	500	30,2
Fehlende Werte		–	–	785	–
*Unterstützung aus privatem Umfeld*	6–17				
Meistens/immer		–	–	2009	88,8
Nie/selten/manchmal		–	–	104	11,2
Fehlende Werte		–	–	781	–
*Allgemeine Gesundheit*	3–17				
Gut (sehr gut/gut)		6554	91,6	–	–
Nicht gut (mittelmäßig–sehr schlecht)		435	8,4	–	–
Fehlende Werte		3	–	–	–
*Allgemeine psychische Gesundheit*	3–17				
Sehr gut (ausgezeichnet/sehr gut)		4691	61,9	–	–
Nicht sehr gut (gut–schlecht)		2293	38,1	–	–
Fehlende Werte		8	–	–	–
*Erhöhter Versorgungs- bzw. Unterstützungsbedarf*	3–17				
Nein		6321	89,6	–	–
Ja		611	10,4	–	–
Fehlende Werte		60	–	–	–
*Teilnahme an schulischen Sport-AGs in den letzten 4 Wochen*	5–15				
Ja		2463	57,2	–	–
Nein		1935	42,8	–	–
Fehlende Werte		2594	–	–	–
*Teilnahme an Sportverein/-kurs in den letzten 4 Wochen*	3–17				
Ja		4213	56,1	–	–
Nein		2321	43,9	–	–
Fehlende Werte		458	–	–	–
*Inanspruchnahme psychosozialer Unterstützungsangebote in den letzten 4 Wochen*	3–17				
Ja		–	–	129	5,0
Nein		–	–	2687	95,0
Fehlende Werte		–	–	78	–

### Statistische Methoden

Aufbauend auf der Beschreibung der beiden Familienformen entlang von Alter, sozialer Lage sowie Belastungen und Ressourcen von Kindern und Jugendlichen zum Ende der Pandemie 2022/2023 werden für die einzelnen Gesundheits-Outcomes gewichtete Prävalenzen stratifiziert nach Familienform (Ein-Eltern- versus Zwei‑Eltern-Haushalt), inklusive der 95 %-Konfidenzintervalle und *p*-Werte des Chi^2^-Tests, berichtet (*Forschungsfrage 1*).

Im zweiten Schritt wurden Prevalence Ratios (PR) mittels Poisson-Regressionen unter Einbezug aller Kontrollvariablen (Modell 1: Geschlecht, Alter, Wohnregion, Untersuchungsjahr) berechnet. Schrittweise erfolgte die Hinzunahme der Mediatorvariablen in die Modelle (Modell 2: Kontrollvariablen + Einkommen, Modell 3: Kontrollvariablen + Bildung). Modell 4 umfasst die PR für die vollständig adjustierten Modelle. Zu allen PR wurden 95 %-Konfidenzintervalle und *p*-Werte bestimmt. Anhand dieser Modelle wird ersichtlich, ob die in der deskriptiven Analyse gefundenen Unterschiede in der Gesundheit und dem Gesundheitsverhalten zwischen den beiden Familienformen sich auch bei Berücksichtigung soziodemografischer und sozialer Determinanten als signifikant darstellen (*Forschungsfrage 2*).

Mit Blick auf *Forschungsfrage 3* wurden für die in der Telefonbefragung erhobenen Outcome-Variablen Poisson-Regressionen mit Interaktionstermen aus Familienform und Einkommen berechnet (adjustiert für Geschlecht, Alter, Wohnregion, Untersuchungsjahr). Als Effektschätzer werden auf Basis der Predicitive Margins vorhergesagte Wahrscheinlichkeiten berichtet, die als adjustierte Prävalenzen zu interpretieren sind. Diese werden inklusive der 95 %-Konfidenzintervalle grafisch dargestellt.

In die multivariaten Modellierungen wurden nur Fälle einbezogen, für die vollständige Angaben zu den Kontroll- und Mediatorvariablen vorliegen.

Als statistisch signifikant wurden *p*-Werte kleiner als 0,05 gekennzeichnet. Die Berechnungen wurden mit einer Design‑, Anpassungs- und Dropout-Gewichtung durchgeführt [32]. Das Designgewicht berücksichtigt die unterschiedliche Auswahlwahrscheinlichkeit der Teilnehmenden, in die Stichprobe gezogen worden zu sein [33]. Bei den 3‑ bis 15-jährigen Teilnehmenden wurde bei der Berechnung der Auswahlwahrscheinlichkeit die Anzahl der Kinder im Haushalt berücksichtigt. Die Anpassungsgewichtung passt die Stichprobe an bekannte Bevölkerungsverteilungen bezüglich Region, Alter, Geschlecht und Bildungsstand der Eltern an. Zur Minimierung von Selektionseffekten wurden für Analysen mit den Online-Daten zusätzlich Dropout-Gewichte auf Basis der Informationen aus der Telefonbefragung berücksichtigt [31]. Die Analysen wurden mit der Statistiksoftware StataSE 17 (StataCorp, College Station, TX, USA) berechnet.

## Ergebnisse

In der KIDA-Studie (telefonischer Befragungsteil) leben 20,6 % der Kinder und Jugendlichen in einem Haushalt mit einem Elternteil (Tab. 2). Tab. 3 beschreibt die soziale Lage von Kindern und Jugendlichen in Abhängigkeit von der Familienform. Bei Familien von Alleinerziehenden ist das Armutsgefährdungsrisiko höher und der höchste Bildungsabschluss im Haushalt niedriger als bei Zwei‑Eltern-Haushalten. Kinder und Jugendliche aus Ein-Eltern-Haushalten waren zudem häufiger durch finanzielle Einschränkungen, Konflikte in der Familie und beengte Wohnverhältnisse belastet. Unterstützung aus dem privaten und dem schulischen bzw. betrieblichen Umfeld erhielten Kinder und Jugendliche aus Ein-Eltern-Haushalten seltener als Heranwachsende, die mit 2 Elternteilen zusammen in einem Haushalt leben.

**Tab. 3:** Soziale Lage von Kindern und Jugendlichen in Ein-Eltern- und Zwei‑Eltern-Haushalten (gewichtete Prävalenzen (in %), 95 %-Konfidenzintervalle, *p*-Werte), Quelle: KIDA

		Ein-Eltern-Haushalt		Zwei‑Eltern-Haushalt		
	*n*	%	95 %-KI	%	95 %-KI	*p*-Wert
*Alter*	6952					*<* *0,001*
3–6 Jahre		18,1	[14,4–22,6]	30,9	[29,1–32,8]	
7–10 Jahre		31,2	[26,8–36,0]	24,6	[23,0–26,2]	
11–13 Jahre		22,7	[19,1–26,8]	19,4	[18,1–20,9]	
14–15 Jahre		15,8	[12,5–19,9]	12,8	[11,6–14,0]	
16–17 Jahre		12,2	[9,3–15,7]	12,3	[10,8–14,0]	
*Haushaltseinkommen (bedarfsgewichtet)*	6952					*<* *0,001*
Niedrig		34,2	[28,7–40,1]	18,0	[16,0–20,1]	
Mittel		61,2	[55,3–66,8]	67,3	[65,0–69,4]	
Hoch		4,6	[3,2–6,6]	14,7	[13,4–16,1]	
*Bildung (höchster Abschluss im Haushalt)*	6926					*<* *0,001*
Niedrig		35,4	[29,3–42,0]	12,8	[10,6–15,4]	
Mittel		49,8	[43,9–55,7]	50,0	[47,8–52,3]	
Hoch		14,7	[12,4–17,6]	37,2	[35,3–39,1]	
*Belastungen (i.* *d. letzten 4 Wochen)*						
Finanzielle Einschränkungen	2790	44,2	[35,2–53,6]	22,8	[19,4–26,5]	*<* *0,001*
Konflikte in der Familie	2789	64,7	[54,0–74,1]	33,4	[29,8–37,2]	*<* *0,001*
Beengte Wohnverhältnisse	2786	23,3	[16,0–32,8]	13,5	[10,8–16,7]	*0,013*
*Unterstützung (i.* *d. letzten 4 Wochen)*						
Aus privatem Umfeld	2109	74,5	[61,1–84,4]	93,0	[89,7–95,3]	*<* *0,001*
Aus schulischem/betrieblichem Umfeld	2105	58,6	[47,2–69,2]	72,9	[68,0–77,4]	*0,014*

*p*-Werte < 0,05 sind *kursiv* hervorgehoben

Nach Familienform stratifizierte Prävalenzen für unterschiedliche Indikatoren der gesundheitlichen Lage sind in Tab. 4 dargestellt (*Forschungsfrage 1*). Die allgemeine Gesundheit wurde für den überwiegenden Teil der Kinder und Jugendlichen als sehr gut oder gut eingeschätzt. Die Prävalenz für eine sehr gute oder gute Gesundheit liegt bei Zwei‑Eltern-Haushalten mit 93,1 % aber signifikant höher als bei Ein-Eltern-Haushalten mit 86,3 %. Bei der allgemeinen psychischen Gesundheit fallen die Unterschiede zwischen den Familienformen noch größer aus. Für 65,8 % der Kinder und Jugendlichen, die mit 2 Elternteilen zusammenleben, schätzten Eltern bzw. Jugendliche die psychische Gesundheit allgemein als ausgezeichnet oder sehr gut ein. Bei den Ein-Eltern-Haushalten trifft dies nur auf 49,2 % zu. Bei Heranwachsenden aus Ein-Eltern-Haushalten findet sich zudem signifikant häufiger ein erhöhter Versorgungs- bzw. Unterstützungsbedarf (16,2 %) als bei Heranwachsenden aus Zwei‑Eltern-Haushalten (8,8 %). Darüber hinaus wurden psychosoziale Beratungs- und Unterstützungsangebote zur besseren Bewältigung der Herausforderungen der Pandemie signifikant häufiger für Kinder oder von den Jugendlichen selbst in Anspruch genommen, wenn sie in einem Ein-Eltern-Haushalt lebten (10,1 %), als dies bei Zwei‑Eltern-Haushalten der Fall war (3,8 %). An außerschulischen Sportangeboten (Sportverein oder -kurs) hatten Kinder und Jugendliche aus Ein-Eltern-Haushalten in den zurückliegenden 4 Wochen signifikant seltener teilgenommen als diejenigen, die mit 2 Elternteilen zusammen in einem Haushalt leben (43,6 % versus 59,6 %). Bei freiwilligen Bewegungs- oder Sport-AGs in der Schule zeigen sich hingegen keine signifikanten Unterschiede in den Teilnahmequoten nach Familienform.

**Tab. 4:** Gesundheit und Gesundheitsverhalten von Kindern und Jugendlichen in Ein-Eltern- und Zwei‑Eltern-Haushalten (gewichtete Prävalenzen (in %), 95 %-Konfidenzintervalle, *p*-Werte), Quelle: KIDA

			Ein-Eltern-Haushalt		Zwei‑Eltern-Haushalt		
		*n*	%	95 %-KI	%	95 %-KI	*p*-Wert
Allgemeine Gesundheit (sehr gut/gut)	Gesamt	6950	86,3	[81,9–89,7]	93,1	[91,9–94,1]	*<* *0,001*
	Mädchen	3317	88,0	[82,4–92,0]	92,5	[90,7–94,0]	*0,041*
	Jungen	3630	84,6	[77,9–89,5]	93,5	[92,0–94,8]	*<* *0,001*
Allgemeine psychische Gesundheit (ausgezeichnet/sehr gut)	Gesamt	6944	49,2	[44,1–54,4]	65,8	[63,8–67,7]	*<* *0,001*
	Mädchen	3313	51,2	[43,6–58,7]	64,9	[62,0–67,7]	*<* *0,001*
	Jungen	3628	47,4	[40,1–54,7]	66,6	[63,8–69,2]	*<* *0,001*
Erhöhter Versorgungs- bzw. Unterstützungsbedarf	Gesamt	6897	16,2	[12,5–20,8]	8,8	[7,7–10,1]	*<* *0,001*
	Mädchen	3293	12,5	[8,6–17,9]	8,6	[7,0–10,6]	0,089
	Jungen	3601	19,8	[14,2–26,8]	9,0	[7,5–10,8]	*<* *0,001*
Teilnahme an Sportverein oder -kurs (i. d. letzten 4 Wochen)	Gesamt	6499	43,6	[38,0–49,4]	59,6	[57,4–61,8]	*<* *0,001*
	Mädchen	3092	41,5	[34,1–49,4]	58,9	[55,9–61,9]	*<* *0,001*
	Jungen	3404	45,6	[38,2–53,0]	60,3	[57,2–63,3]	*<* *0,001*
Teilnahme an Sport-AG in Schule (i. d. letzten 4 Wochen)	Gesamt	4398	54,1	[47,1–60,9]	58,1	[55,4–60,8]	0,286
	Mädchen	2112	50,6	[40,9–60,2]	56,6	[52,9–60,2]	0,255
	Jungen	2284	57,5	[48,1–66,3]	59,6	[55,8–63,2]	0,679
Inanspruchnahme psychosozialer Angebote (i. d. letzten 4 Wochen)	Gesamt	2811	10,1	[6,1–16,3]	3,8	[2,3–6,1]	*0,004*

*p*-Werte < 0,05 sind *kursiv* hervorgehoben

In Tab. 5 werden die Prevalence Ratios (PR) der schrittweisen Modellierung dargestellt. Wie Tab. 3 zu entnehmen ist, zeigen sich zwischen den Familienformen große Unterschiede in Hinblick auf Einkommen und Bildung, weshalb für diese Variablen in der multivariaten Modellierung adjustiert wurde (*Forschungsfrage 2*). Die Ergebnisse von Modell 1 zeigen, dass auch bei Kontrolle für Alter, Geschlecht, Wohnregion und Untersuchungsjahr die anhand der gewichteten Prävalenzen beobachteten Unterschiede zwischen den Familienformen bestehen bleiben. Die nachfolgenden Modelle verdeutlichen, dass die Effektstärken bei Berücksichtigung des höchsten Bildungsabschlusses sowie des Einkommens des Haushalts zwar insgesamt geringer ausfallen als in Modell 1, aber – mit Ausnahme der Teilnahme an Sport-AGs in der Schule und der Inanspruchnahme psychosozialer Angebote – weiterhin signifikant sind. Bei der Inanspruchnahme psychosozialer Angebote muss die geringe Fallzahl berücksichtigt werden.

**Tab. 5:** Gesundheit und Gesundheitsverhalten von Kindern und Jugendlichen in Ein-Eltern-Haushalten im Vergleich zu Zwei‑Eltern-Haushalten (Prevalence Ratios, 95 %-Konfidenzintervalle, *p*-Werte; Referenzgruppe: Zwei‑Eltern-Haushalte; Kontrollvariablen: Geschlecht, Alter, Wohnregion, Untersuchungsjahr), Quelle: KIDA

		Modell 1		Modell 2		Modell 3		Modell 4	
		Adjustiert für Kontrollvariablen		Adjustiert für Haushaltseinkommen + Kontrollvariablen		Adjustiert für höchste Bildung im Haushalt + Kontrollvariablen		Vollständig adjustiert	
	*n*	PR [95 %-KI]	*p*-Wert	PR [95 %-KI]	*p*-Wert	PR [95 %-KI]	*p*-Wert	PR [95 %-KI]	*p*-Wert
Allg. Gesundheit (sehr gut/gut)	6907	0,93	*0,002*	0,94	*0,004*	0,95	*0,022*	0,95	*0,023*
		[0,89–0,97]		[0,90–0,98]		[0,91–0,99]		[0,91–0,99]	
Allg. psychische Gesundheit (ausgezeichnet/sehr gut)	6904	0,78	*<* *0,001*	0,81	*<* *0,001*	0,81	*<* *0,001*	0,82	*0,001*
		[0,70–0,87]		[0,72–0,90]		[0,73–0,90]		[0,74–0,92]	
Erhöhter Versorgungs- bzw. Unterstützungsbedarf	6858	1,71	*<* *0,001*	1,64	*0,001*	1,49	*0,011*	1,48	*0,011*
		[1,27–2,31]		[1,22–2,19]		[1,10–2,02]		[1,10–2,00]	
Teilnahme an Sportverein oder -kurs (i. d. letzten 4 Wochen)	6463	0,71	*<* *0,001*	0,74	*<* *0,001*	0,80	*0,001*	0,81	*0,002*
		[0,62–0,81]		[0,65–0,85]		[0,70–0,91]		[0,71–0,92]	
Teilnahme an Sport-AG in Schule (i. d. letzten 4 Wochen)	4386	0,95	0,453	0,95	0,425	0,92	0,257	0,92	0,263
		[0,83–1,08]		[0,83–1,08]		[0,81–1,06]		[0,81–1,06]	
Inanspruchnahme psychosozialer Angebote (i. d. letzten 4 Wochen)	2805	2,68	*0,012*	2,20	0,080	2,01	0,183	1,89	0,136
		[1,24–5,80]		[0,91–5,32]		[0,72–5,61]		[0,82–4,38]	

*p*-Werte < 0,05 sind *kursiv* hervorgehoben

**Abb. 1:**
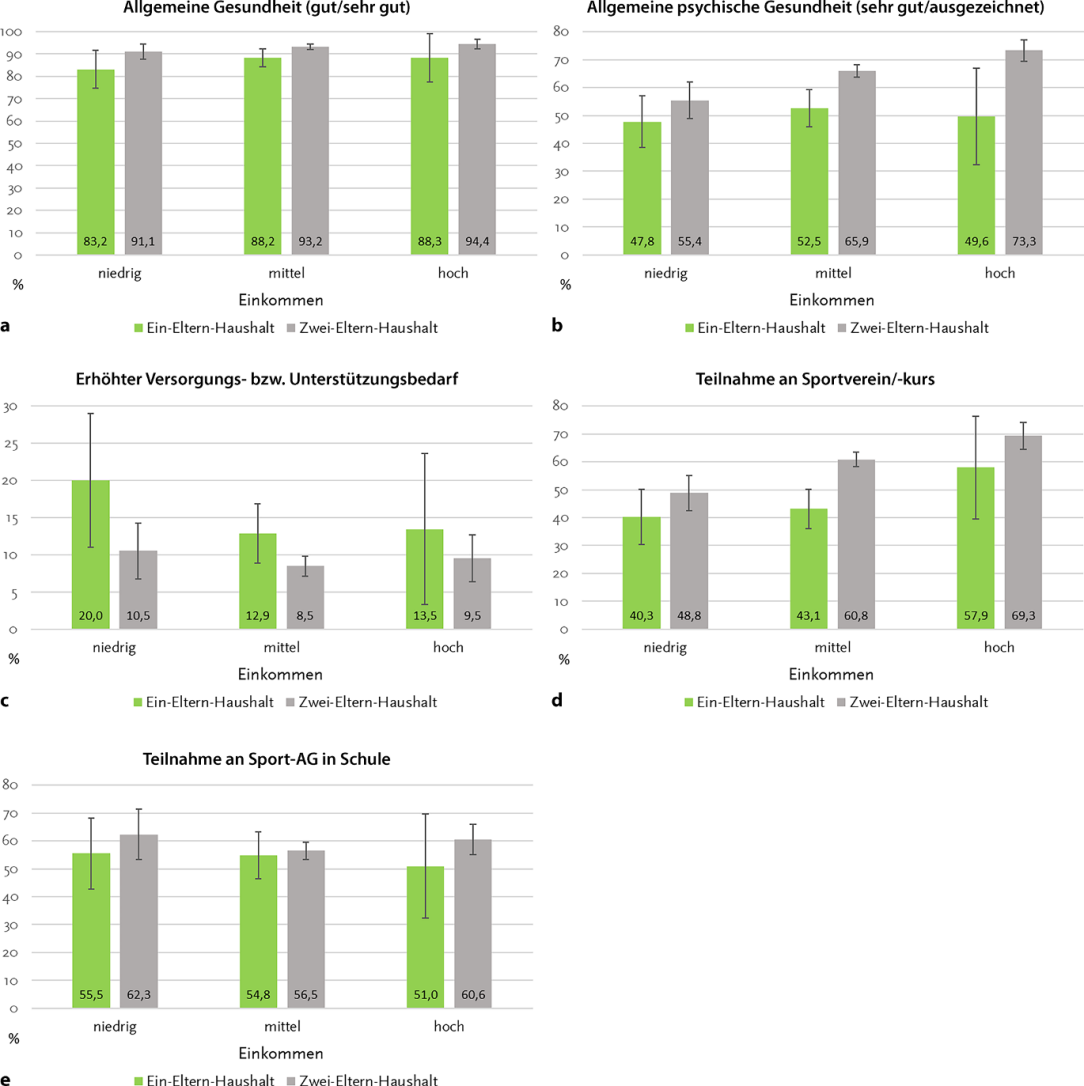
Gesundheit und Gesundheitsverhalten von Kindern und Jugendlichen in Ein-Eltern- und Zwei‑Eltern-Haushalten, stratifiziert nach Haushaltseinkommen (Poisson-Regressionen mit Interaktionen zwischen Familienform und Einkommen, vorhergesagte Wahrscheinlichkeiten (in %), 95 %-Konfidenzintervalle, alle Modelle adjustiert für Geschlecht, Alter, Wohnregion, Untersuchungsjahr). Quelle: KIDA. **a** Allgemeine Gesundheit (gut/sehr gut), **b** allgemeine psychische Gesundheit (sehr gut/ausgezeichnet), **c** erhöhter Versorgungs- bzw. Unterstützungsbedarf, **d** Teilnahme an Sportverein/-kurs, **e** Teilnahme an Sport-AG in Schule

Abb. 1 verdeutlicht, dass die Zusammenhangsmuster zwischen Familienform und Gesundheit nur in geringem Maße mit dem Einkommen variieren (*Forschungsfrage 3*); die Interaktionen fallen für alle Gesundheits-Outcomes statistisch nicht signifikant aus. Mit Blick auf die allgemeine psychische Gesundheit fällt jedoch auf, dass sich bei Kindern und Jugendlichen aus Zwei‑Eltern-Haushalten ein signifikanter Einkommensgradient zeigt, nicht aber bei Kindern und Jugendlichen aus Ein-Eltern-Haushalten. In Ein-Eltern-Haushalten wird in allen Einkommensgruppen nur etwa für eines von 2 Kindern oder Jugendlichen eine sehr gute oder ausgezeichnete psychische Gesundheit berichtet. Während dies für armutsgefährdete Kinder und Jugendliche aus Zwei‑Eltern-Haushalten ebenso zutrifft, wird die allgemeine psychische Gesundheit bei Heranwachsenden aus Zwei‑Eltern-Haushalten mit mittlerem oder hohem Einkommen signifikant häufiger als sehr gut oder ausgezeichnet eingeschätzt als bei Gleichaltrigen aus Ein-Eltern-Haushalten. Auch bei der Teilnahme an Sportvereinen und -kursen besteht bei Kindern und Jugendlichen aus Zwei‑Eltern-Haushalten ein signifikanter Einkommensgradient. Bei Heranwachsenden aus Ein-Eltern-Haushalten zeigt sich zwischen der niedrigen und mittleren Einkommensgruppe hingegen kein Unterschied in der Teilnahme an Sportkursen oder -vereinen.

## Diskussion

Studien stellten bereits zu Beginn der COVID-19-Pandemie eine starke psychosoziale Belastung von Kindern und Jugendlichen aus Ein-Eltern-Haushalten fest [18, 20]. Die KIDA-Ergebnisse zeigen, dass eine hohe Belastung von Heranwachsenden aus Ein-Eltern-Haushalten durch finanzielle Einschränkungen, Konflikte in der Familie und beengte Wohnverhältnisse auch am Ende der Pandemie noch gegeben ist, wobei die hier dargestellten Belastungen nicht ausschließlich pandemiebedingt sind, sondern sich für Ein-Eltern-Haushalte auch schon vor der Pandemie beobachten ließen [34]. Gerade weil Heranwachsende aus Ein-Eltern-Haushalten stärker psychosozial belastet waren als Gleichaltrige aus Zwei‑Eltern-Haushalten, wäre eine Unterstützung im schulischen oder betrieblichen Umfeld wichtig. Die KIDA-Ergebnisse zeigen jedoch, dass mehr als 40 % der Kinder von Alleinerziehenden im schulischen oder betrieblichen Umfeld nicht ausreichend Unterstützung erfuhren.

Mit Blick auf die gesundheitliche Lage lässt sich in der KIDA-Studie für Kinder und Jugendliche aus Ein-Eltern-Haushalten eine durch Eltern bzw. Jugendliche selbst als weniger gut eingeschätzte allgemeine sowie psychische Gesundheit als bei Gleichaltrigen aus Zwei‑Eltern-Haushalten beobachten. Für die psychische Gesundheit deckt sich dies mit Ergebnissen anderer Studien aus der Zeit vor [10–12, 14, 15] und während der Pandemie [18, 21]. Zu beachten ist dabei, dass die psychische Gesundheit in den Studien mit anderen Instrumenten gemessen wurde, sodass keine direkte Vergleichbarkeit möglich ist.

Bezüglich des allgemeinen Gesundheitszustands fallen die hier berichteten Unterschiede nach Familienform deutlich geringer aus als bei der psychischen Gesundheit. Dies könnte darin begründet liegen, dass in die Einschätzung der allgemeinen Gesundheit auch körperliche Aspekte eingehen, für die nur vereinzelt Unterschiede nach Familienform beschrieben werden [16]. Während einige Studien ebenfalls Unterschiede in der allgemeinen Gesundheit zwischen den Familienformen zuungunsten der Ein-Eltern-Haushalte berichten [16, 35, 36], zeigen sich bei anderen Studien keine Unterschiede [12, 37].

Analog zu einer stärkeren Beeinträchtigung der allgemeinen und psychischen Gesundheit zeigt sich in der KIDA-Studie häufiger ein erhöhter Versorgungs- und Unterstützungsbedarf bei Kindern und Jugendlichen aus Ein-Eltern-Haushalten. In internationalen Studien (z. B. [38]) werden für den Gesamt-CSHCN(Children with Special Health Care Needs)-Screener ebenfalls höhere Ausprägungen bei Heranwachsenden aus Ein-Eltern-Haushalten berichtet. Auch wenn für Deutschland keine Studien vorliegen, lässt sich anhand anderer Indikatoren ein größerer Versorgungsbedarf bei Heranwachsenden aus Ein-Eltern-Haushalten erkennen. So wurde alleinerziehenden Müttern Anfang 2022 häufiger von ÄrztInnen oder PsychotherapeutInnen eine psychotherapeutische Behandlung ihres Kindes empfohlen [18]. In einer Umfrage unter AOK-Versicherten [18] gaben Anfang 2022 3 von 4 Alleinerziehenden an, dass sie sich professionelle Unterstützung wünschen, um ihrem Kind die Bewältigung der pandemiebedingten Belastungen zu erleichtern. Von Alleinerziehenden wurde insbesondere Unterstützung durch SchulpsychologInnen und -sozialarbeiterInnen sowie durch Sportvereine und KinderärztInnen gewünscht. Sie formulierten zudem größeren Unterstützungsbedarf durch Kinder- und JugendpsychiaterInnen/-psychotherapeutInnen, Erziehungsberatungsstellen und das Jugendamt [18] als Zwei‑Eltern-Haushalte.

Zum höheren Versorgungs- und Unterstützungsbedarf passt die in der KIDA-Studie häufiger berichtete Inanspruchnahme psychosozialer Unterstützungs- und Beratungsangebote durch Alleinerziehende und ihre Kinder, die für die vorpandemische Zeit auch in anderen Studien beobachtet wurde [39]. Bei differenzierter Betrachtung verschiedener Beratungs- und Unterstützungsangebote werden bei Lux et al. [40] in Abhängigkeit von der Familienform unterschiedliche Inanspruchnahmeprofile sichtbar: So nutzten Alleinerziehende mit Kleinkindern vor der Pandemie häufiger Familien- oder Erziehungsberatungsstellen, Angebote des Jugendamtes sowie eine längerfristig aufsuchende Betreuung durch eine Familienhebamme [40]. Eltern-Kind-Gruppen wurden hingegen seltener von Alleinerziehenden und Angebote in Familien- oder Stadtteilzentren von allen Familien etwa gleich häufig in Anspruch genommen [40]. Die KIDA-Daten legen zudem nahe, dass zum Ende der Pandemie Kinder- und Jugendlichenpsychotherapien häufiger von Ein-Eltern- als von Zwei‑Eltern-Haushalten in Anspruch genommen wurden, während sich für sozialpädiatrische Zentren keine Unterschiede in der Inanspruchnahme nach Familienform zeigen [31]. Inwieweit der Versorgungsbedarf durch die Inanspruchnahme gedeckt wurde, kann mit den Daten der KIDA-Studie nicht analysiert werden. Es ist jedoch anzunehmen, dass aufgrund von Zugangsbarrieren oder Versorgungsengpässen Angebote – trotz vorhandenen Bedarfs – nicht in Anspruch genommen wurden.

Ein mit Blick auf Prävention und Gesundheitsförderung wichtiges Ergebnis ist, dass Kinder und Jugendliche aus Ein-Eltern-Haushalten seltener an Angeboten in Sportvereinen oder an Sportkursen außerhalb der Schule teilnahmen, aber mit freiwilligen schulischen Bewegungs- und Sportangeboten annähernd gleich häufig wie Gleichaltrige aus Zwei‑Eltern-Haushalten erreicht wurden. Dass Heranwachsende, die mit einem Elternteil zusammenleben, signifikant seltener Sportvereine und -kurse besuchen, wurde bereits in anderen Studien beobachtet [16, 41]. Ein möglicher Grund wird darin gesehen, dass es Alleinerziehenden logistisch im Alltag oftmals schwerer fällt, eine regelmäßige Teilnahme ihrer Kinder an diesen Angeboten sicherzustellen [41]. Dies ist insbesondere bei einer Erwerbstätigkeit und wenig flexiblen Arbeitszeiten der Fall. Da ein hoher Anteil von Ein-Eltern-Haushalten armutsgefährdet ist, ist die Zahlung eines Mitgliedsbeitrags aus finanziellen Gründen oftmals erschwert [42]; eine Inanspruchnahme von Förderleistungen etwa durch Mittel aus dem Bildungs- und Teilhabepaket ist mitunter schambelastet [43]. Anders als für die Teilnahme an außerschulischen Sportkursen konnten für außerunterrichtliche Bewegungs- und Sportangebote in der Schule keine Studien gefunden werden, die wie in unserer Studie Unterschiede nach Familienform untersuchen. Hier besteht weiterer Forschungsbedarf.

Mit Blick auf die soziale Lage von Ein-Eltern-Haushalten konnten in dieser Studie in Teilen Mediationseffekte gefunden werden. So werden die beschriebenen Unterschiede in der Gesundheit und dem Gesundheitsverhalten zwischen den Familienformen bei Kontrolle für soziodemografische und sozioökonomische Faktoren zwar schwächer, bleiben aber signifikant. Sie lassen sich somit nicht gänzlich durch sozioökonomische Unterschiede erklären. Ergebnisse anderer Studien divergieren sehr stark in Abhängigkeit vom betrachteten Gesundheitsindikator [10, 12, 15] und dem Alter der Heranwachsenden [10].

Daneben zeigt sich, dass die beschriebenen Zusammenhänge zwischen Familienform und Gesundheit bzw. Gesundheitsverhalten bei Kindern und Jugendlichen kaum mit dem Haushaltseinkommen variieren. Somit haben die Familienform und das Haushaltseinkommen unabhängig voneinander einen additiven Effekt auf die Gesundheit und das Gesundheitsverhalten von Kindern und Jugendlichen, interagieren aber kaum miteinander. Dieses Ergebnis findet sich auch bei Grüning Parache et al. [10] für emotionale und Verhaltensauffälligkeiten sowie die gesundheitsbezogene Lebensqualität. Die Annahme, dass sich in armutsgefährdeten Familien die hohe Belastung von Ein-Eltern-Haushalten in der Pandemie besonders ungünstig auf die Gesundheit und das Gesundheitsverhalten auswirkte, konnte mit dieser Studie nicht belegt werden. Vielmehr zeigt sich, dass in Ein-Eltern-Haushalten – unabhängig vom Einkommen des Haushalts – nur bei etwa der Hälfte der Heranwachsenden die allgemeine psychische Gesundheit als sehr gut oder ausgezeichnet eingeschätzt wird. Bezüglich der psychischen Gesundheit, aber in der Tendenz auch bei der Teilnahme an Sportvereinen oder -kursen spielt somit weniger die benachteiligte finanzielle Situation von Ein-Eltern-Haushalten eine Rolle als vermutlich eher begrenzte zeitliche und soziale Ressourcen der Eltern oder spezifische psychosoziale Belastungen von Trennungsfamilien. Zur Absicherung dieser Hypothese bedarf es allerdings weiterer Forschung.

Die KIDA-Studie weist einige Limitationen auf [44]. Aufgrund der Art des Stichprobenzugangs sind die Ergebnisse nicht für Deutschland repräsentativ. Selektionseffekte ergeben sich aus einer geringeren Teilnahmebereitschaft von Eltern aus Haushalten mit niedriger Bildung sowie von Jugendlichen. Zudem ist nicht auszuschließen, dass stark belastete Eltern und Jugendliche seltener an der KIDA-Studie teilgenommen haben. Die Kombination aus Telefon- und Online-Befragung bedeutet zudem einen Wechsel des Erhebungsmodus und geht mit einer deutlich geringeren Fallzahl in der Online-Befragung einher. Für online erhobene Variablen (hier: Inanspruchnahme psychosozialer Angebote) ist es daher nicht möglich, neben der Familienform auch noch für das Einkommen zu stratifizieren. Darüber hinaus ist bei der Interpretation der Ergebnisse zu berücksichtigen, dass für die 3‑ bis 15-Jährigen Elternangaben und für die 16- bis 17-Jährigen Selbstangaben einbezogen wurden, die divergieren können [45]. Sowohl die allgemeine Gesundheit als auch die allgemeine psychische Gesundheit wurden jeweils nur mittels eines Items im Eltern- bzw. Selbstbericht erhoben. Es handelt sich hierbei um keine Diagnosen, sondern um subjektive Gesundheitseinschätzungen der Eltern bzw. Jugendlichen. Die eingesetzten Fragen gelten jedoch als valide Erhebungsinstrumente, die mit klinischen Diagnosen und objektivierbaren Outcomes korrelieren [46, 47]. In weiterführenden Analysen sollten die hier vorgestellten Zusammenhänge zwischen den subjektiven Globalmaßen für Gesundheit und der Familienform auch für spezifische Diagnosen (z. B. ADHS (Aufmerksamkeitsdefizit-/Hyperaktivitätsstörung)) bzw. symptombasierte Gesundheitsindikatoren analysiert werden. Bei der KIDA-Studie handelt es sich zudem um eine Querschnittsbefragung, die keine Aussagen zur Richtung des Zusammenhangs zwischen Familienform und Gesundheit bzw. Gesundheitsverhalten (Kausalität versus Selektion) erlaubt. Mit Blick auf die Familienform ermöglicht die KIDA-Studie lediglich eine Differenzierung in Ein-Eltern- und Zwei‑Eltern-Haushalte. Für Ein-Eltern-Haushalte liegen keine Information dazu vor, ob der Lebensmittelpunkt des Kindes in dem Haushalt des befragten Elternteils oder ggf. in dem des anderen Elternteils liegt oder das Kind etwa zu gleichen Teilen bei beiden Elternteilen lebt. Somit kann die Diversität der Familienformen [4, 5] hier nur bedingt abgebildet werden.

Ob gesundheitliche Beeinträchtigungen von Kindern und Jugendlichen aus Ein-Eltern-Haushalten in oder durch die Pandemie größer geworden sind, wurde in diesem Beitrag nicht analysiert, da keine mit denselben Instrumenten bzw. für dieselben Altersgruppen publizierten Daten aus der Zeit direkt vor der Pandemie vorliegen. Für einzelne Indikatoren, für die für bestimmte Altersgruppen vergleichbare Daten aus der KiGGS-Studie (Studie zur Gesundheit von Kindern und Jugendlichen in Deutschland) vorliegen, können im nächsten Schritt Trendanalysen berechnet werden. Darüber hinaus wäre es wünschenswert, eine eigene Kinder- und Jugendstichprobe in das Anfang 2024 gestartete bevölkerungsbezogene Panel „Gesundheit in Deutschland“ [48] zu integrieren, um zukünftig Längsschnitt- und Trendanalysen durchführen zu können.

## Fazit

Insgesamt zeigen sich zum Ende der Pandemie ähnliche Zusammenhangsmuster zwischen der Familienform und den verschiedenen gesundheitsbezogenen Outcomes wie vor der Pandemie: Kinder und Jugendliche aus Ein-Eltern-Haushalten sind oftmals stärker sozial belastet und in Teilen häufiger gesundheitlich beeinträchtigt als Kinder und Jugendliche aus Zwei‑Eltern-Haushalten. Kinder und Jugendliche aus Ein-Eltern-Haushalten sind somit eine wichtige Zielgruppe für Gesundheitsförderung und Prävention [49]. Insbesondere Einrichtungen der Kindertagesbetreuung, Schulen und Stadtteile/Kommunen stellen wichtige Lebenswelten dar, in denen ein förderndes Umfeld für eine gesunde Entwicklung geschaffen werden kann und in denen Kinder und Jugendliche aus Ein-Eltern-Haushalten niedrigschwellig und stigmatisierungsfrei erreicht werden können [49, 50].

Mit Blick auf die gesundheitlichen Folgen der Pandemie für Kinder und Jugendliche empfiehlt der „ExpertInnenrat der Bundesregierung zu COVID-19“ Maßnahmen, die nicht nur pandemiebedingte Probleme kompensieren, sondern vorrangig darauf zielen, bereits zuvor bestehende soziale und gesundheitliche Ungleichheiten nachhaltig zu vermindern [51]. Hierzu bedarf es aus Sicht des ExpertInnenrats psychosozialer und psychotherapeutischer Angebote mit niedrigschwelliger schulischer Anbindung und zum anderen erweiterte Jugendhilfemaßnahmen, einschließlich Schulsozialarbeit und Stärkung der stadtteilbezogenen offenen Jugendarbeit in sozial benachteiligten Wohnquartieren [51]. Bujard et al. empfehlen zudem den Ausbau von Familienbildungs- und Familienberatungseinrichtungen [19]. Laut der „Interministeriellen Arbeitsgruppe Kindergesundheit“ sollen Angebote zur Stärkung der Gesundheit von Kindern und Jugendlichen für alle zugänglich und deshalb an Regelstrukturen angebunden sein, sodass sich Schule als zentraler Ort anbietet [50]. Sie benennt in diesem Zusammenhang explizit die Stärkung der Resilienz und mentalen Gesundheit von SchülerInnen sowie die Förderung von Sport und Bewegung an und im Umfeld von Schulen [50]. Dass Kinder und Jugendliche aus Ein-Eltern-Haushalten – wie dargestellt – etwa gleich häufig an freiwilligen Bewegungs- und Sportangeboten in der Schule teilnahmen wie Gleichaltrige aus Zwei‑Eltern-Haushalten, stützt diese Vorschläge und zeigt das Potenzial von Sport- und Freizeitangeboten an oder in Kooperation mit Schulen und Kitas zur Verringerung von gesundheitlichen Ungleichheiten im Kindes- und Jugendalter auf.

Im Sinne des Ansatzes der familiären Gesundheitsförderung [52] ist die Gesundheit von Kindern und Jugendlichen systemisch mit Blick auf die Familie als Ganzes zu verstehen. Daher sollten auch die Rahmenbedingungen für die ganze Familie gesundheitsförderlich ausgestaltet werden. Hierzu zählen auf gesamtgesellschaftlicher Ebene insbesondere Maßnahmen zum Abbau des strukturell bedingten hohen Armutsrisikos sowie die Schaffung von Rahmenbedingungen, die es Alleinerziehenden ermöglichen, ausreichend Zeit mit ihren Kindern zu verbringen [53].

Evaluationsstudien sollten gezielt für Alleinerziehende und ihre Kinder analysieren, welche Maßnahmen der Gesundheitsförderung sich als wirksam erweisen [54].

## Data Availability

Die AutorInnen geben an, dass für die den Ergebnissen zugrunde liegenden Daten einige Zugangsbeschränkungen gelten. Der Datensatz kann nicht öffentlich zugänglich gemacht werden, da die Einwilligung (Informed Consent) der Studienteilnehmenden die öffentliche Bereitstellung der Daten nicht abdeckt. Der minimale Datensatz, der den Ergebnissen zugrunde liegt, ist im Forschungsdatenzentrum des Robert Koch-Instituts archiviert und kann von Forschenden mit begründeter Anfrage eingesehen werden. Der Datenzugriff ist vor Ort im Secure Data Center des Forschungsdatenzentrums des Robert Koch-Instituts möglich. Anfragen können per E‑Mail an fdz@rki.de gestellt werden.

## Footnotes

1 Der Begriff Alleinerziehende wird in diesem Beitrag in Anlehnung an die Definition des Statistischen Bundesamtes [55] für Mütter und Väter verwendet, die ohne PartnerIn mit mindestens einem minderjährigen Kind zusammen in einem Haushalt leben, unabhängig davon, ob das oder die Kinder ggf. zeitweise im Haushalt des anderen Elternteils leben.
